# Characterization of Human Adrenal Steroidogenesis During Fetal Development

**DOI:** 10.1210/jc.2018-01759

**Published:** 2018-12-21

**Authors:** Cecilie Melau, John Erik Nielsen, Hanne Frederiksen, Karen Kilcoyne, Signe Perlman, Lene Lundvall, Lea Langhoff Thuesen, Kristine Juul Hare, Anna-Maria Andersson, Rod T Mitchell, Anders Juul, Anne Jørgensen

**Affiliations:** 1 Department of Growth and Reproduction, Rigshospitalet, University of Copenhagen, Copenhagen, Denmark; 2 International Center for Research and Research Training in Endocrine Disruption of Male Reproduction and Child Health, Rigshospitalet, Copenhagen, Denmark; 3 MRC Centre for Reproductive Health, The Queen’s Medical Research Institute, University of Edinburgh, Edinburgh, United Kingdom; 4 Department of Gynaecology, University Hospital of Copenhagen (Rigshospitalet), Copenhagen, Denmark; 5 Department of Obstetrics and Gynaecology, Hvidovre University Hospital, Hvidovre, Denmark

## Abstract

**Context:**

The endocrine function of human fetal adrenals (HFAs) is activated already during first trimester, but adrenal steroidogenesis during fetal life is not well characterized.

**Objective:**

This study aimed to investigate HFA steroidogenesis by analyzing adrenal glands from first and second trimesters.

**Design and Setting:**

Male and female HFA from gestational weeks (GWs) 8 to 19 were examined, including a total of 101 samples from 83 fetuses.

**Main Outcome Measure(s):**

Expression level of steroidogenic genes and protein expression/localization were determined by quantitative PCR and immunohistochemistry, respectively, and intra-adrenal steroid levels were quantified by LC-MS/MS.

**Results:**

Transcriptional levels of *StAR, CYP11A1, CYP17A1, CYP21A2, CYP11B1/2,* and *SULT2A1* were significantly higher in second trimester compared to first trimester (*P* < 0.05), whereas expression levels of *3β-HSD2* and *ARK1C3* were unaltered between GWs 8 and 19. All investigated steroidogenic proteins were expressed in a distinct pattern throughout the investigated period, with most enzymes expressed primarily in the fetal zone, except 3*β*-HSD1/2, which was expressed mainly in the definitive zone. Abundant steroidogenic enzyme expression was reflected in overall high intra-adrenal tissue concentrations of mineralocorticoids, glucocorticoids, and androgens; cortisol was the most abundant (1071 to 2723 ng/g tissue), and testosterone levels were the lowest (2 to 14 ng/g tissue).

**Conclusions:**

The expression profiles of HFA steroidogenic enzymes are distinct from first to second trimester, with no major differences between male and female samples. Intra-adrenal steroid hormone concentrations confirm that cortisol is produced throughout first and second trimesters, suggesting continued regulation of the hypothalamus-pituitary-adrenal axis during this entire period.

## Teaser

Investigation of steroidogenic enzyme expression and steroid hormone secretion demonstrates that human fetal adrenal steroidogenesis is actively regulated throughout the first and second trimesters.

The morphology of the human fetal adrenal (HFA) gland differs from that of the adult organ. Thus, the morphology of the HFA gland is established around gestational week (GW) 7, where the adrenal cortex has differentiated from adrenogenital primordial cells into a distinct morphology consisting of two separate zones, a thin outer definitive zone (DZ) and an inner fetal zone (FZ) that accounts for 80% to 90% of the adrenal tissue (1). During the second trimester, an additional transitional zone (TZ) develops at the interface between the DZ and FZ, after which the morphology of the HFA gland is unchanged until birth (1).

Activation of the hypothalamus-pituitary-adrenal (HPA) hormone axis is essential for adrenal steroidogenic enzyme expression (2). Thus, activation of the adrenal melanocortin receptor 2 (MC2R) through binding of pituitary-secreted ACTH is necessary for the endocrine capacity of the HFA gland (1, 3, 4). The FZ (and later the TZ) is described as the most steroidogenic active zone, expressing Steroidogenic acute regulatory protein (StAR), cytochrome P450 11A1 (CYP11A1), cytochrome P450 17A1 (CYP17A1), cytochrome P450 21A2 (CYP21A2), cytochrome P450 11B (CYP11B1/CYP11B2), and Sulfotransferase Family 2A Member 1 (SULT2A1) from around GW 7 (4–6). Throughout gestation, CYP17A1 and SULT2A1 are exclusively localized to the FZ and TZ (1, 7–9), whereas CYP11A1, CYP21A2, and StAR are also expressed in the DZ from around GW 23 (7, 8, 10, 11).

3*β*-hydroxysteroid dehydrogenase type 2 (3*β*-HSD2) mediates the adrenal *de novo* synthesis of *Δ*^4^ steroid hormones. Because the adrenal expression of *3β-HSD1* is very low compared with that of *3β-HSD2* (4, 12), the detection of 3*β*-HSD1/2 in this study is thought to represent mainly expression of 3*β*-HSD2. In contrast to the other HFA steroidogenic enzymes, 3*β*-HSD2 is expressed mainly in DZ cells from GWs 7 to 10 (3, 4, 7, 8, 11). However, a recent study showed a continued 3*β*-HSD2 expression throughout the second trimester (13). This finding suggests that in addition to its role in the regulation of fetal adrenal cortisol-mediated negative feedback on the HPA axis to minimize adrenal androgen synthesis during the critical time window of sex differentiation (4, 14–16), the 3*β*-HSD2 enzyme also may act as a negative regulator throughout the second trimester.

HFA steroidogenesis is tightly regulated throughout the first and second trimesters, which is crucial because the adrenal steroid hormones affect the overall intrauterine endocrine environment from early fetal development. Elevated levels of HFA androgens can be a consequence of dysregulated adrenal steroid pathways, in which imbalanced steroidogenesis can cause masculinization of the external genitals in female fetuses with congenital adrenal hyperplasia (CAH) (17). However, HFA steroidogenesis is not well characterized during fetal development, as previous studies investigating the steroidogenic function have focused on either the first or the second/third trimester only, characterizing selected adrenal steroidogenic enzymes. This study therefore aimed to collect detailed expression data of all the classic steroidogenic enzymes and determine the intra-adrenal steroid levels from the first- and second-trimester HFAs in one inclusive study. Thus, this study examined both HFA tissue steroid levels and gene and protein expression of steroidogenic enzymes throughout first- and second-trimester fetal development.

## Material and Methods

### Collection of human fetal adrenals and ethical approvals

HFA tissue from the first trimester (GWs 8 to 12) and second trimester (GWs 14 to 19) were isolated from material available following elective surgical termination of pregnancy at Copenhagen University Hospital (Rigshospitalet) and Hvidovre Hospital, Denmark, as well as the Royal Infirmary of Edinburgh, United Kingdom, and the Human Developmental Biology Resource, United Kingdom. A total of 101 HFA samples collected from 83 individual fetuses were used in this study, as two adrenals from the same fetus were occasionally used in different analyses. The study was approved by the Danish regional ethics committee (H-1-2012-007) and the UK Lothian Research Ethics committee (LREC08/S1101/1). Woman gave their informed written and oral consent. None of the terminations were for reasons of fetal abnormality, and all fetuses appeared morphologically normal. Fetal age was determined by scanning crown-rump length and confirmed by evaluation of foot length (18). Hence, fetal age is about 2 weeks greater than in studies reporting days/weeks after conception. After dissection, adrenal tissue samples were either snap frozen at −80°C or fixed immediately in 4% buffered formalin. Fetal sex determination was based on PCR using specific primers targeting *SRY* (Table 1). DNA for sex determination was extracted from fetal limb tissue and isolated with NucleoSpin Genomic DNA as described by the manufacturer (Macherey-Nagel, Düren, Germany). PCR cycle conditions were as follows: one cycle of 3 minutes at 95°C, 25 cycles of 30 seconds at 95°C, 1 minute at 60°C, 1.5 minutes at 65°C, and one cycle of 5 minutes at 72°C.

**Table 1.** Primers Used for RT-PCR and Quantitative PCR

Gene	HGNC ID	Forward Primer	Reverse Primer	Application Size (bp)
*StAR*	11359	CACCCCTAGCACGTGGATTA	CTTGGTTGCTAAGGATGCCC	152
*CYP11A1*	2590	ATAAACCGACTCCACGTTGC	ACAATGGCTGGCTAAACCTG	134
*CYP17A1*	2593	GAGTTTGCTGTGGACAAGGG	CGCTGGATTCAAGAAACGCT	117
*3β-HSD2*	5218	CAGGCTCTTTTCAGGAATGG	CTTGGACAAGGCCTTCAGAC	117
*CYP21A2*	2600	GAGTTCTGTGAGCGCATGAG	GAATCACGTCCACAATTTGGAT	205
*CYP11B1/2*	2591/2	CTTCCACTACACCATAGAAGCCAGC	CCTCAAAGTGCTCCTTCCACAC	200
*ARK1C3*	386	GGAGGCCATGGAGAAGTGTAAGGA	CCAGAGCACTATAGGCAACCAGAAC	215
*SULT2A1*	11458	ACAGGACACAGGAAGAACCATAGAG	CTTCAGCTTGGGCCACTGTGAA	230
*MC2R*	6930	ACATGGGCTATCTCAAGCCAC	TCCAGATGACCGTAAGCACCA	204
*RPS20*	10405	AACAAGCCGCAACGTAAAATC	ACGATCCCACGTCTTAGAACC	166
*SRY*	11311	GAATATTCCCGCTCTCCGGA	GCTGGTGCTCCATTCTTGAG	470

All primers are shown in 5′ to 3′ direction.

Abbreviations: *ARK1C3,* aldo-keto reductase family 1 member C3; HGNC, HUGO Gene Nomenclature Committee.

### Gene expression

Total RNA was extracted from one frozen HFA gland in samples from GWs 8 to 12 (11 male and 11 female, collected from 22 fetuses), whereas half of a frozen HFA gland was used in samples from GWs 14 to 19 (9 male and 8 female, collected from 17 fetuses) and isolated using the NucleoSpin RNA II purification kit according to the manufacturer’s instructions (Macherey-Nagel, Düren, Germany). cDNA was synthesized using a dT20 primer and random hexamers. Real-time polymerase chain reaction (RT-PCR) was performed using specific primers targeting preselected mRNAs. All primers were designed to span intron-exon boundaries with optimal annealing temperatures of ∼62°C, comparable primer length, and CG contents (Table 1). All amplicons were initially verified by sequencing (Eurofins MWG GmbH, Ebersberg, Germany), and primer amplification efficiency and detectable dynamic range of all primer sets were validated before the analysis of the HFA samples. RT-PCR cycle conditions were as follows: one cycle of 3 minutes at 95°C, 40 cycles of 30 seconds at 95°C, 1 minute at 62°C, 1 minute at 72°C, and one cycle of 5 minutes at 72°C. Quantitative RT-PCR analysis was performed in triplicate using Brilliant II SYBR Green qPCR Master Mix (Agilent Technologies, Santa Clara, CA). Changes in gene expression were quantified using the 2^−^*^ΔΔ^*^Ct^ method (19). Expression levels were normalized to the reference gene, *RPS20*, and calculated as a ratio with male samples GWs 8 to 9 set to 1 in respective samples.

### Immunohistochemistry

Adrenal tissue from GWs 8 to 12 (13 male and 13 female, collected from 26 fetuses) and GWs 14 to 19 (10 male and 6 female, collected from 16 fetuses) were used for immunohistochemistry (IHC). Formalin-fixed adrenal tissues were dehydrated, paraffin embedded, and sectioned (4 µm) using standard procedures. Primary antibodies, dilutions, and retrieval buffers are listed in Table 2. IHC staining was initially conducted according to a standard protocol as described previously (20). Subsequently, the protocol was modified to include antigen retrieval by pressure cooker as previous described (21). In brief, tissue sections were subjected to heat-induced antigen retrieval buffer in a pressure cooker, and endogenous peroxidase was blocked with 3% (v/v) H_2_O_2_ in MeOH for 30 minutes. Between each step, sections were washed in Tris-buffered saline. Sections were incubated in 5% BSA (w/v) in horse serum (20% v/v) ImmPRESS (Vector Laboratories, Burlingame, CA) and Tris-buffered saline (80% v/v) or 0.5% milk powder for 30 minutes depending on optimization for each antibody. Sections were subsequently incubated overnight with primary antibody diluted in serum at 4°C in a humidified chamber followed by 1 hour at room temperature. Sections were then incubated for 30 minutes with the appropriate ImmPRESS HRP (peroxidase, Vector Laboratories) secondary antibody diluted in normal serum. Visualization was performed using ImmPACT AEC peroxidase substrate (Vector Laboratories, Burlingame, CA). Included negative controls replaced the primary antibody with dilution buffer only, none of which showed any staining. All sections were counterstained with Mayer hematoxylin before mounting with Aquatex (Merck, Damstadt, Germany).

**Table 2.** Antibodies, Dilutions, and Retrieval Buffers Used

Antibody	Dilution	Retrieval Buffer	Species	Supplier	Number
CYP11A1	1:10,000	TEG	Rabbit	Sigma	HPA016436
CYP17A1	1:1500	CIT	Rabbit	Abcam	Ab134910
3*β*-HSD1/2	1:6000	TEG	Rabbit	Gift from Ian Mason	—
CYP21A2	1:6000	TEG	Goat	Santa Cruz Biotechnology	Sc-48466
CYP11B1	1:1000	TEG	Mouse	Santa Cruz Biotechnology	Sc-374096
SULT2A1	1:6000	CIT	Rabbit	Sigma	HPA041487
MC2R	1:800	TEG	Rabbit	Santa Cruz Biotechnology	Sc-13107

Antigen retrieval was conducted by pressure cooking of the sections in indicated retrieval buffer for 30 minutes in a decloaking chamber. TEG buffer: 10 mM Tris, 0.5 mM EGTA, pH 9.0; citrate (CIT) buffer: 10 mM, pH 6.0.

### Quantification of stained cells

Two independent investigators evaluated all stainings. Sections were first investigated manually on a Nikon Microphot-FXA microscope; subsequently, slides were scanned on a NanoZoomer 2.0 HT (Hamamatsu Photonics, Herrsching am Ammersee, Germany) and analyzed using NDP view software, version 1.2.36 (Hamamatsu Photonics). The intensity of immunoreactivity was classified according to a predefined scoring system: ++, strong staining in all cells of a given type in the sample; ++/+, strong staining prevalent, but some weakly stained cells also visible; ++/−, strong staining present, but negative cells also present; +/++, majority of cells weakly stained, but some strong staining present; +/++/−, heterogeneous pattern with a mixture of strongly positive, weakly stained, and negative cells; + (1), weak staining overall or (2) strong staining in a small number of cells; +/−, weak staining in limited areas; and −/+, weak staining in single cells.

### Quantification of steroid hormones

Tissue samples from intact HFA glands from GWs 8 to 12 (10 male and 10 female, collected from 20 fetuses) and halved HFA glands from GWs 14 to 19 (9 male and 8 female, collected from 17 fetuses) were included for the intra-adrenal steroid analysis by LC-MS/MS measurements. Tissue samples were weighed (1.7 to 43 mg wet weight) and ground in 1 mL 80% (v/v) MeOH. The homogenate was transferred to glass tubes evaporated to almost dryness under an N_2_ stream. Internal standard stock solution (5 to 500 ng/mL in 100 µL) was added to each sample pellet as previously described (22), and 375 µL 1M ammonium acetate buffer (pH, 5.5) was subsequently added. Next, 2 mL Heptan/Ethylacetat 3:2 (v/v) was added, and samples were transferred to Eppendorf tubes. Samples were then shaken for 15 minutes followed by 10 minutes of centrifugation at 2000*g* (4°C). Each tube was transferred to a dry ice bath (dry ice pills in ethanol, 99%) for a few minutes to freeze the aqueous phase, followed by decantation of the organic phase to a new glass tube. The organic phase was evaporated to dryness under a stream of N_2_, and finally, the steroids were resolved in an appropriate amount of 50% (v/v) MeOH (tissue GWs 8 to 12: 100 µL; tissue GWs 14 to 19: 200 µL) for LC-MS/MS analysis as previously described (22). All samples were measured in one single batch, which included standards for calibration curves, unknown samples, and two blanks and for method control; three unspiked human serum pool samples and three serum pool samples spiked with low and high levels, respectively.

### Statistical analysis

Quantitative PCR and LC-MS/MS data were statistically analyzed for age- and sex-specific differences. Age differences were tested by the nonparametric Mann-Whitney *U* test in which the HFA age groups GWs 10 to 12, GWs 14 to 16, and GWs 17 to 19 were compared with male GWs 8 to 9. Sex differences were also tested by the nonparametric Mann-Whitney *U* test within each age group. *P* < 0.05 was considered statistically significant.

## Results

### Gene expression patterns of adrenal steroidogenic enzymes

**Figure 1.**
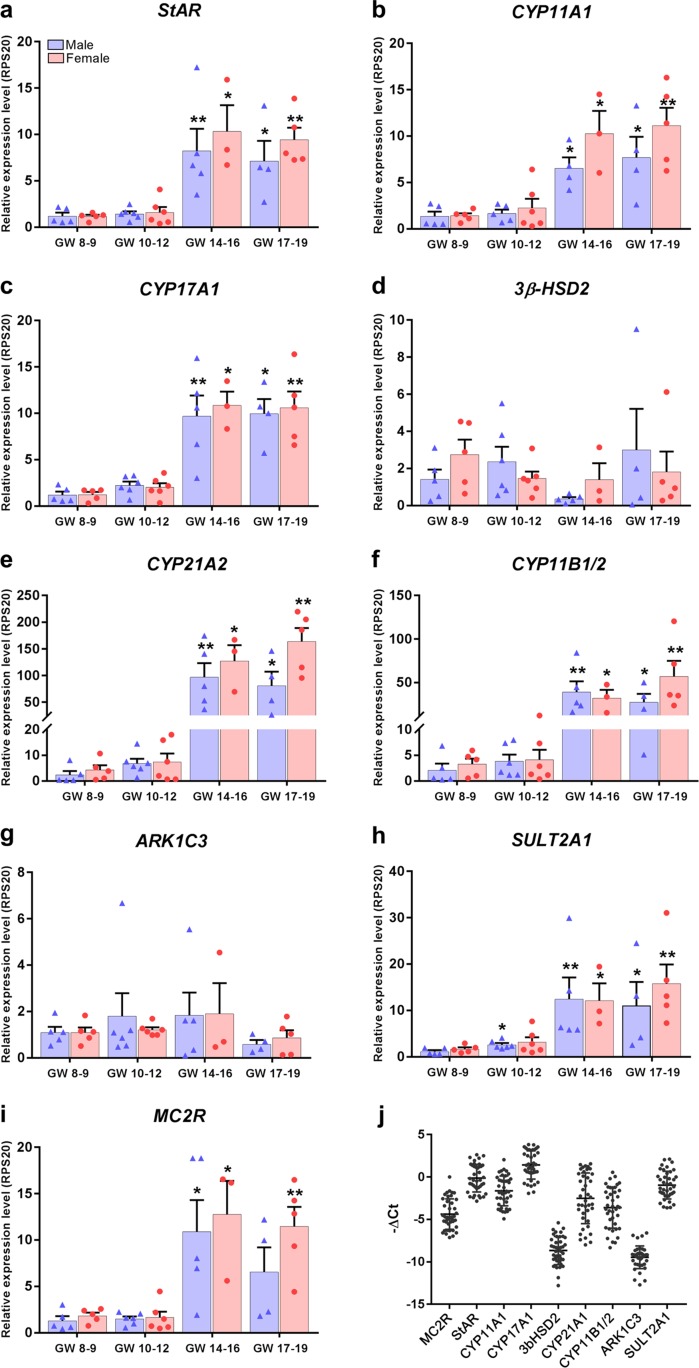
Gene expression level of human fetal adrenal steroidogenic enzymes during the first and second trimesters. (a‒i) Quantitative reverse transcription polymerase chain reaction analysis of a range of steroidogenic-associated enzymes and receptors in male and female human fetal adrenal samples divided into four age groups: GWs 8 to 9, GWs 10 to 12, GWs 14 to 16, and GWs 17 to 19. Expression is relative to the reference gene *RPS20*. The expression level is set to 1 in male GWs 8 to 9 samples. In total, 39 adrenal samples were used. Bars represent mean ± SEM with individual data points shown as blue triangles and red circles for male and female samples, respectively; n = 3 to 6. Differences in age compared with male GWs 8 to 9 are indicated as significantly different: *P < 0.05; **P < 0.01. (j) Overall human fetal adrenal transcript levels from first- and second-trimester samples; n = 39. Data points represent −*Δ*Ct values (relative to the housekeeping gene *RPS20*) of investigated gene transcript levels in individual adrenal samples (male and female). Error bars represent mean ± SD. *ARK1C3*, aldo-keto reductase family 1 member C3.

The selected steroidogenic enzymes were expressed in all investigated HFA glands at the transcriptional level. Gene expression patterns were investigated separately in male and female samples, which were divided into four age groups: GWs 8 to 9, GWs 10 to 12, GWs 14 to 16, and GWs 17 to 19. Expression levels were calculated as a ratio of levels relative to male GWs 8 to 9 (reference value set to 1). No sex differences were observed in the transcription levels of the examined steroidogenic enzymes or in the transcription levels of the ACTH receptor, *MC2R*, throughout the investigated developmental period (Fig. 1). During the second trimester, the expression level of *StAR, CYP11A1, CYP17A1, SULT2A1,* and *MC2R* increased approximately 10-fold, whereas the expression level of *CYP21A2* and *CYP11B1/2* increased even further, by >50-fold and >20-fold, respectively (Fig. 1e and 1f). Only two of the investigated steroidogenic enzymes were constitutively expressed throughout the first and second trimesters, namely *3β-HSD2* and aldo-keto reductase family 1 member C3 (*ARK1C3;* also known as 17*β*HSD3) (Fig. 1d and 1g). In addition, the absolute gene expression levels of *3β-HSD2* and *ARK1C3* were lower than those of the remaining steroidogenic enzymes (Fig. 1j).

### Distribution of steroidogenic enzymes in the HFA cortex

**Figure 2.**
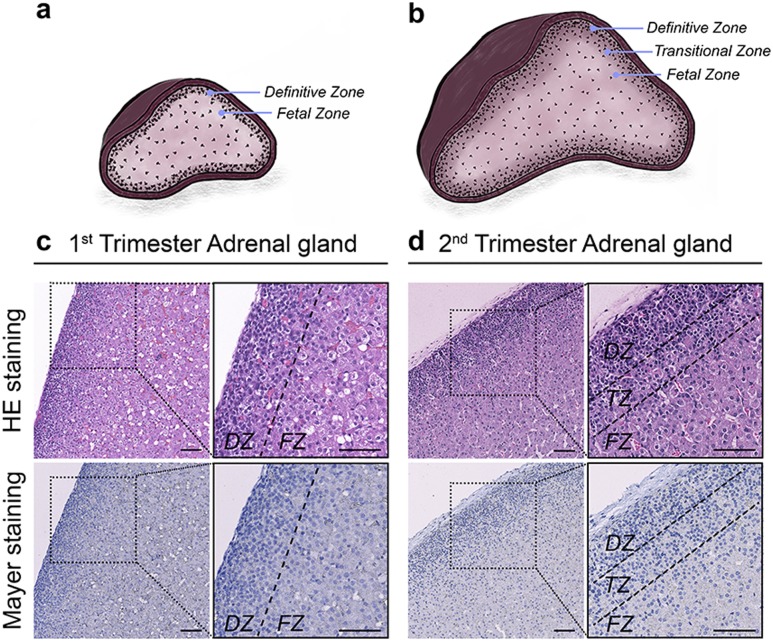
Human fetal adrenal gland morphology and zonation. Schematic illustrations of the distinct morphological zonation in (a) first-trimester human fetal adrenals (GWs 8 to 12) and (b) second-trimester human fetal adrenals (GWs 14 to 19) viewed as a cross section of the gland. (c) Hematoxylin and eosin (HE) and Mayer stainings of a first-trimester human fetal adrenal gland. (d) HE and Mayer stainings of a second-trimester human fetal adrenal gland. Scale bar = 100 µm.

The morphology of fixed adrenal samples included in the study was thoroughly examined. All samples appeared morphologically normal, containing distinct zones in accordance with the gestational age of the developing HFA gland (Fig. 2). Sections were stained with hematoxylin and eosin as well as Mayer hematoxylin. The DZ consisted of small cells, whereas FZ cells were larger, with a high content of cytoplasm (Fig. 2c). As expected, the TZ was detected in second-trimester samples and consisted of a mixture of small distinct DZ cells and larger distinct FZ cells (Fig. 2d), as previous described (1).

**Figure 3.**
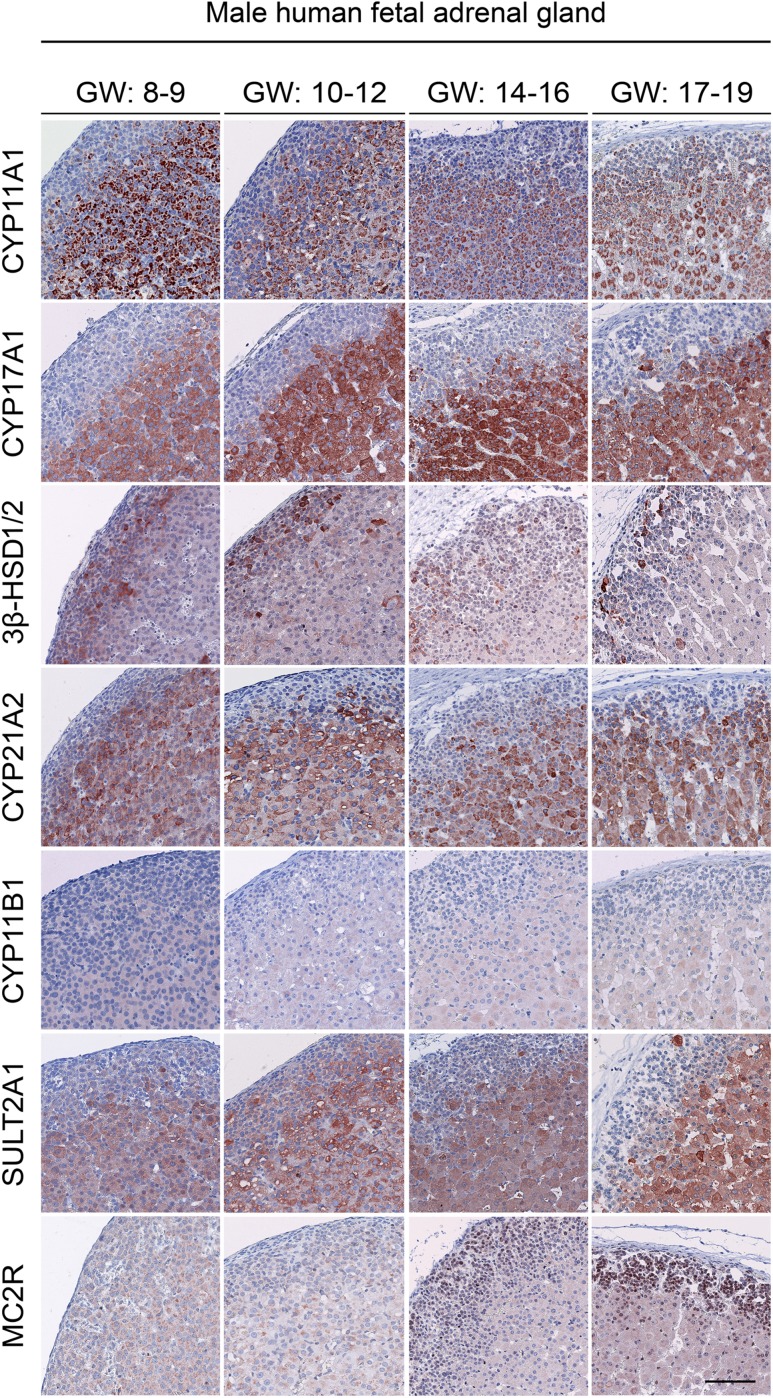
Expression of steroidogenic enzymes in first- and second-trimester human adrenal glands. Immunohistochemical staining of serial sections for CYP11A1, CYP17A1, 3*β*-HSD1/2, CYP21A2, CYP11B1, and SULT2A1 (steroidogenic proteins) as well as MC2R (ACTH receptor) in male samples are shown. No differences were observed between male and female samples within a given gestational group; therefore, only male samples are shown. Scale bar = 100 µm.

The expression profiles of steroidogenic proteins were analyzed using IHC on fixed adrenal samples. No differences between male and female samples were observed regarding the level of expression and the cell type‒specific enzyme localization. Therefore, only male samples are shown for the age groups GWs 8 to 9, GWs 10 to 12, GWs 14 to 16, and GWs 17 to 19 in Fig. 3. Adrenal zone‒specific protein expression, localization, and level of expression were evaluated according to a predefined scale and are summarized in Table 3.

**Table 3.** Analysis of Adrenal Zone-Specific Protein Localization and Level of Expression

	DZ				FZ				C
GW: Enzyme	8–9	10–12	14–16	17–19	8–9	10–12	14–16	17–19	17–19
CYP11A1	+ (2)	+ (2)	+ (2)	+/++/−	++	++/+	++/+	++/+	
CYP17A1	−/+	+ (2)	+ (2)	+ (2)	++/+	++/+	++ (TZ), ++/+ (FZ)	++ (TZ), ++/+ (FZ)	
SULT2A1	+ (2)	+ (2)	+ (2)	+ (2)	++/+	++/+	++/+	++/+	
MC2R	+ (1)	+/++	+/++, +/++/− (n)	+ (1), ++/− (n)	+/++	+/++	+/++, −/+(n)	+/++, +/++/− (n)	++/− (n)
CYP11B1	+/−	−/+	−/+	−/+	+/−	+/−	+ (1)	+ (1)	
CYP21A2	−/+	+ (2)	+ (2)	+ (2)	+/++	++/+	++/+	++/+	
3*β*-HSD1/2	++/−	++/−	+/++/−	+/++/−	+ (2)	+ (2)	+ (2)	+ (2)	

n = 6*−*15 adrenals within each gestational group. TZ staining is indicated when the TZ protein expression differed from the FZ expression in the second-trimester tissue (GWs 14*−*19). Scale: ++, strong staining in all cells of a given type in the sample; ++/+, strong staining prevalent, but some weakly stained cells also visible; ++/−, strong staining present, but negative cells also present; +/++, majority of cells weakly stained, but some strong staining present; +/++/−, heterogeneous pattern with a mixture of strongly positive, weakly stained, and negative cells; + (1), weak staining overall or (2) strong staining in a small number of cells; +/−, weak staining in limited areas; −/+, weak staining in single cells.

Abbreviations: C, capsule; n, nuclear staining.

Most of the investigated steroidogenic enzymes were abundantly expressed in the FZ throughout development of both the first and second trimesters (Fig. 3). More specifically, CYP11A1, CYP21A2, and SULT2A1 were abundantly expressed in the cytoplasm of first-trimester FZ cells and weakly expressed in a limited number of DZ cells toward the late first trimester and throughout the second trimester. CYP17A1 showed a similar expression and localization pattern, although CYP17A1 was expressed specifically in the FZ except for a few strongly positive cells scattered within the DZ in both first- and second-trimester samples. During the second trimester, protein expression of CYP17A1 increased around the TZ as it decreased in the cells of the inner FZ. In contrast, 3*β*-HSD1/2 was the only observed steroidogenic enzyme frequently expressed in a limited number of DZ cells and in only a very few FZ cells. During the second trimester, 3*β*-HSD1/2 was still abundantly expressed, though only in a subpopulation of DZ and FZ cells compared with first-trimester samples.

Like the steroidogenic enzymes, the ACTH receptor MC2R was also detected throughout the investigated period of fetal development. More specifically, weak cytoplasmic MC2R expression was detected throughout the first and second trimesters, but nuclear MC2R expression was also occasionally found in the late first trimester and throughout the second trimester. Therefore, the MC2R expression patterns observed in this study should be interpreted with caution because the unexpected nuclear expression (23) is most likely an artifact of the antibody.

### HFA steroid tissue concentrations

The endocrine activity of the HFA was further evaluated by determination of the intra-adrenal steroid concentrations. Thus, steroids were extracted from adrenal gland tissue followed by LC-MS/MS determination of mineralocorticoid, glucocorticoid, and androgen levels. Samples were divided according to sex and age (as described previously). All investigated mineralocorticoids, glucocorticoids, and androgen metabolites were detected in HFA tissue from GWs 8 to 19.

**Figure 4.**
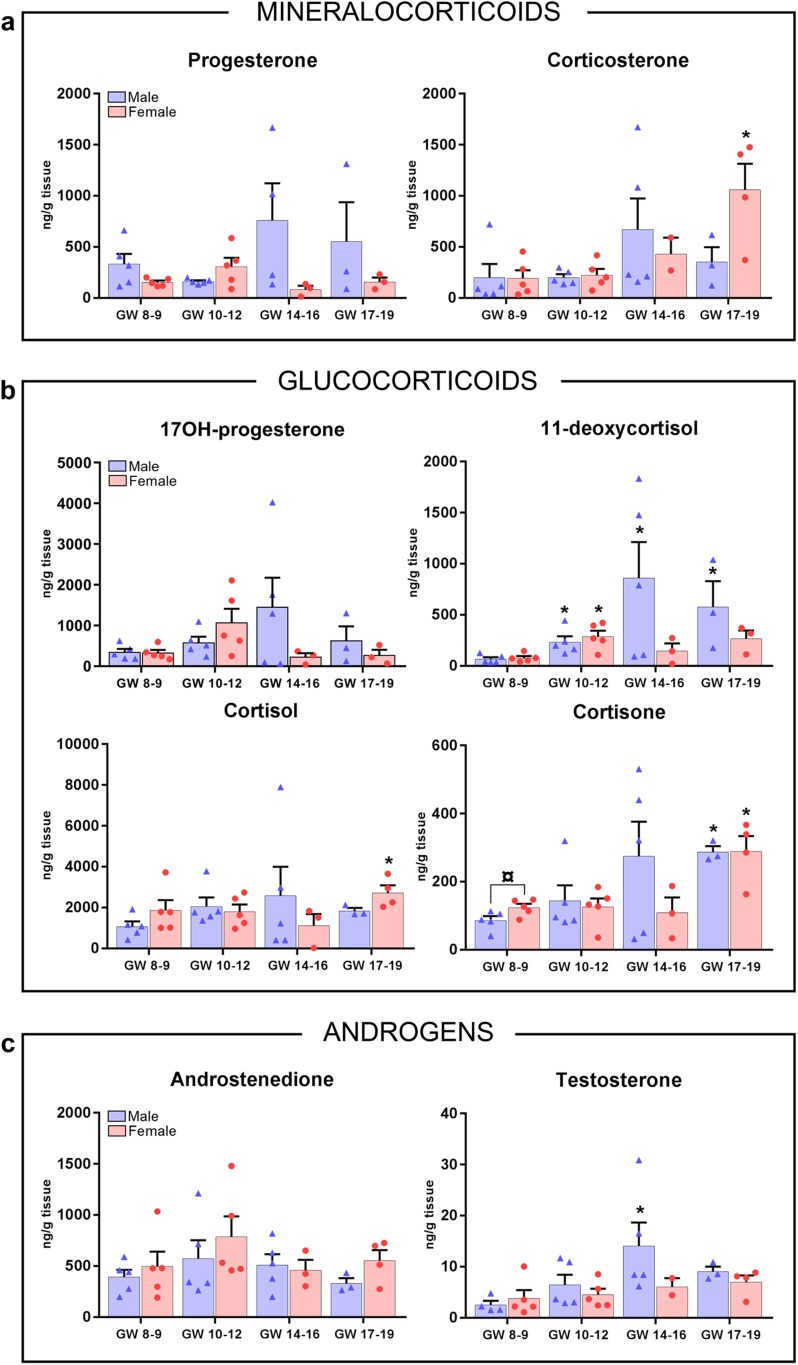
Tissue levels of steroids in first- and second-trimester human adrenals. (a‒c) LC-MS/MS measurements (ng/g, wet tissue) of adrenal steroid hormones were determined from male and female adrenal tissue extracts. Bars represent mean ± SEM; individual data points shown as blue triangles and red circles represent male and female samples, respectively; n = 3 to 5. Differences in age compared with male GWs 8 to 9 are indicated as significantly different: *P < 0.05. Differences between sexes within the same age group are indicated as significantly different ¤*P* < 0.05.

Measured concentrations of the mineralocorticoid metabolites progesterone and corticosterone were, in general, equivalent, with no significant difference in relation to either gestational age or sex. The only exception was a significant age-related increase in female corticosterone levels at GWs 17 to 19 compared with male tissue concentrations at GWs 8 to 9 (Fig. 4a). The intra-adrenal levels of the glucocorticoid 17OH-progesterone were unaltered over the investigated developmental period, with no detectable differences between male and female samples. In contrast, in 11-deoxycortisol, cortisol and cortisone, a significant increase in intra-adrenal steroid concentrations was found at GWs 17 to 19 compared with male GWs 8 to 9 (Fig. 4b). For 11-deoxycortisol, a significant increase in intra-adrenal steroid concentrations was further observed at GWs 10 to 12 and GWs 14 to 16, the latest only in male samples. The only observed sex-specific difference was found in intra-adrenal cortisone concentrations, which were significantly higher in female samples at GWs 8 to 9 than in age-matched males (Fig. 4b). Interestingly, cortisol levels were the highest of all measured intra-adrenal steroid hormones throughout the investigated developmental period. Hence, cortisol tissue concentrations were approximately twofold higher than those of 17OH-progesterone and 11-deoxycortisol and approximately 10-fold higher than that of cortisone (Fig. 4b).

Similar to the trend for the majority of measured steroid hormones, the adrenal tissue concentration of androgens was relatively constant throughout GWs 8 to 19, with a significant increase in testosterone levels found only in male GWs 14 to 16 samples compared with male GWs 8 to 9 tissue concentrations (Fig. 4c). The adrenal conversion of androstenedione to testosterone appeared to be limited, as tissue concentrations of androstenedione were approximately 80-fold higher than concentrations of testosterone (Fig. 4c).

## Discussion

**Figure 5.**
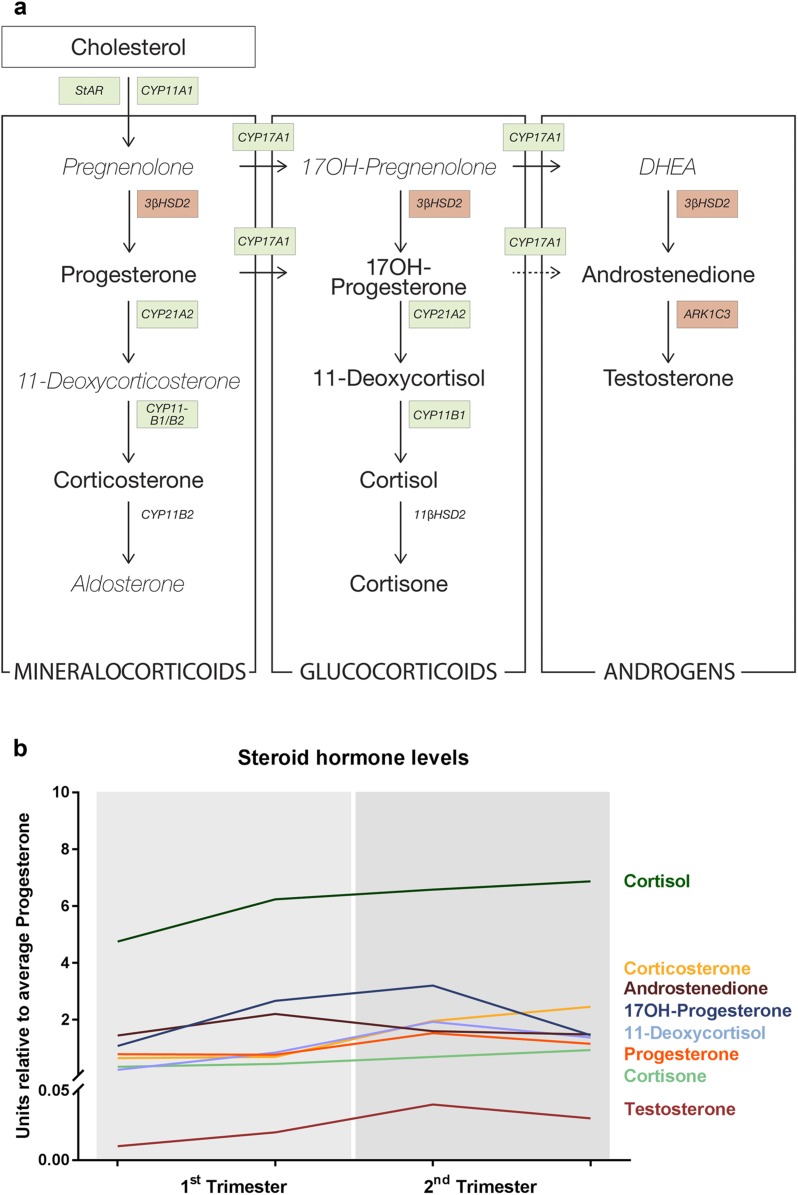
Overview of human fetal adrenal steroidogenic development. (a) Schematic overview of adrenal steroidogenesis. Gene transcripts of enzymes significantly upregulated in the second trimester are shown in green boxes, with unaltered gene transcripts shown in red boxes. Measured steroid hormones are shown in boldface font. (b) Overview of steroid hormone levels relative to progesterone levels throughout the investigated developmental period. Data are shown as mean values of fetal adrenal samples (for SEM values, see data in Fig. 4). DHEA, dehydroepiandrosterone.

This study investigated the adrenal steroidogenic expression pattern during the first and second trimesters of human fetal development. HFA glands function as active endocrine organs from early fetal development, which was confirmed by this study, demonstrating expression of all investigated steroidogenic enzymes both at the gene and the protein levels in all investigated samples from GWs 8 to 19. The distinct morphological expression pattern of the investigated enzymes is in accordance with those from previous studies focusing on either first- or second-trimester human adrenals (4, 7, 8, 10, 11, 13, 14). Thus, our data support the prominent hypothesis of the FZ, and later in development also the TZ, being the main site of *de novo* steroid synthesis in the HFA. Interestingly, the transcription of steroidogenic genes appeared to be regulated differently, with unaltered expression of *ARK1C3* and *3β-HSD2* during the investigated period, whereas the expression of the other steroidogenic enzymes (*StAR, CYP11A1, CYP17A1, CYP21A2, CYP11B1/2,* and *SULT2A1)* and the ACTH receptor *MC2R* was significantly increased in the second trimester (Fig. 5a). The observed increases in gene expression levels in the second trimester were not evident at the protein level, presumably because of the lack of sensitivity of IHC to detect differences in expression levels. However, the genes upregulated during the second trimester were also the steroidogenic enzymes that showed the most abundant immunostaining in the FZ, while the low constitutively expressed 3*β*-HSD1/2 was detected only in a small subpopulation of cells located mainly in the DZ. Furthermore, the upregulation in gene expression for the majority of the investigated steroidogenic enzymes was not reflected in higher tissue concentrations of steroidogenic hormones, which overall did not differ between the first and second trimesters (Fig. 5b). It remains to be examined whether the second-trimester transcriptional upregulation of steroidogenic enzymes is reflected in steroid levels secreted by the HFA to the fetal circulation, which was not possible to determine in this study.

The constitutive expression of 3*β*-HSD1/2 throughout the first and second trimesters of human fetal development in male and female HFA samples contrasts with the previously reported transient expression profile (3, 4, 7, 8, 11). Thus, our results demonstrate that 3*β*-HSD1/2 is highly expressed in a subpopulation of DZ cells from GWs 8 to 19, which is in accordance with recently published data (13). Although the percentage of 3*β*-HSD1/2‒positive cells in the DZ and FZ appeared to decrease during the second trimester, this study reports a small number of 3*β*-HSD1/2‒positive FZ cells in second-trimester fetuses up to GW 19. In general, the small number of 3*β*-HSD1/2‒positive cells compared with the abundant expression of the majority of the adrenal steroidogenic enzymes is consistent with reported low *3β-HSD2* transcript levels in this study, as well as in previous reports (3, 4, 11, 13). The immediate imbalance between low 3*β*-HSD1/2 expression and high tissue concentrations of 3*β*-HSD2‒catalyzed *Δ*^4^ steroids—progesterone, 17OH-progesterone, and androstenedione—was previously suggested to be the result of either circulating placental progesterone or the transfer of intermediates of the steroid pathways between the adrenal zones (13). The demonstration by double immunofluorescence staining in second-trimester HFAs that CYP11A1 and CYP21A2 colocalize in only a limited number of FZ cells (13) could support the hypothesis of transport of steroidogenic intermediates between the adrenal zones. Moreover, the presence of high levels of intra-adrenal androstenedione in the second trimester determined in this study favors 3*β*-HSD2‒dependent *de novo* synthesis, as 17OH-progesterone is a poor substrate for human CYP17A1 (C17,20 lyase) (24), indicating that adrenal androstenedione is not likely generated from placental progesterone.

The HFAs produced androgens throughout the first and second trimesters in both male and female samples. The detection of high HFA tissue concentrations of androstenedione and low levels of testosterone indicate that this step-in steroidogenesis is tightly regulated. The low intra-adrenal testosterone levels detected from GWs 8 to 19 are in accordance with previous studies that have examined both fetal adrenal tissue concentrations and secreted levels in organ cultures (3, 4, 13). Furthermore, the low tissue concentrations of testosterone are in accordance with the relatively low transcriptional expression level of *ARK1C3,* which mediates adrenal *de novo* testosterone synthesis. The expression level of *ARK1C3* in first-trimester samples detected in this study supports previous investigations (3, 4), whereas *ARK1C3* expression levels, to our knowledge, have not been investigated previously in second-trimester HFA tissue. Consequently, the significant increase in male testosterone levels at GWs 14 to 16 compared with levels in male GWs 8 to 9 is inconsistent with the observed overall unaltered expression level of *ARK1C3,* suggesting that testosterone levels are not regulated solely via gene transcription. The observed peak in male testosterone around GWs 14 to 16 is not significantly different from levels in the female GWs 14 to 16 age group, and it is likely that adrenal testosterone production is considerably lower than the contribution from the fetal testis (25). Moreover, this study reported that androstenedione is the most abundant *Δ*^4^ adrenal androgen synthesized by the classic steroidogenic pathway throughout the first and second trimesters in HFA, which contrasts with a previously reported higher adrenal androgen production during the first trimester (13).

The HFA produces cortisol throughout the first and second trimesters, indicating continued regulation of the HPA axis during this entire period. In line with this finding is the detection of cytoplasmic MC2R expression from GW 8, which is in accordance with findings of previous studies (3, 4). However, because of the unexpected additional nuclear expression of MC2R observed in this study, these results should be interpreted with caution; MC2R is known to be expressed in the cytoplasm (at the endoplasmic reticulum) until it is translocated with MRAP to the plasma membrane to mediate ACTH signaling (23).The abundant HFA tissue concentrations of cortisol detected in this study further support the recent hypothesis that cortisol-mediated negative feedback of the HPA axis is not restricted to the first trimester but extends through the second trimester (13). Detection of intra-adrenal cortisol in the second trimester is consistent with the observed expression of 3*β*-HSD1/2 and is in accordance with a previous study that detected cortisol in HFA tissue from the second trimester (13), although the cortisol concentrations measured in the current study are approximately 10-fold higher than the concentrations previously reported. During GWs 8 to 14, cortisol-mediated negative feedback on the HPA axis is thought to be crucial because this period is the window of development in which the sex-specific differentiation of gonads and external genitalia occurs (15, 16). Hence, dysregulated adrenal steroidogenesis, which results in an imbalance with an excess of adrenal androgens and reduced cortisol in this time window, can cause virilization of female genitalia in fetuses with CAH (17). Because adrenal androgens can be converted to estrogens by the placental aromatase (CYP19A1) later in pregnancy, the adrenal secretion of androgens during the second trimester is thought to have less effect on genital differentiation (15, 16). However, the observed abundant concentration of cortisol throughout the first and second trimesters raises new questions about whether alterations in cortisol production can still affect virilization of fetuses with CAH during the second trimester or may possibly influence other events during human fetal development.

This study provides detailed characterizations of both male and female adrenal endocrine functions during the first and second trimesters of human fetal development. It is evident from the gene and protein expression patterns of steroidogenic enzymes, as well as the steroid measurements in tissues, that the HFA functions as an active steroidogenic organ from early development by producing high levels of mineralocorticoids, glucocorticoids, and androgens. Even from GW 8, we report a distinct expression pattern for the investigated adrenal steroidogenic enzymes, with a major increase in gene expression in second-trimester samples for the majority of steroidogenic enzymes, with the exception of the unaltered expression of *3β-HSD2* and *ARK1C3*. On the basis of the intra-adrenal steroid hormone concentrations, we found that androstenedione is the most abundant *Δ*^4^ adrenal androgen synthesized via the classic steroidogenic pathway throughout the first and second trimesters. In addition, this study confirms that cortisol is produced throughout the first and second trimesters, suggesting continued regulation of the HPA axis during this entire period.

## Abbreviations:

3*β*-HSD2: 3*β*-hydroxysteroid dehydrogenase type 2
ARK1C3: aldo-keto reductase family 1 member C3
CAH: congenital adrenal hyperplasia
CYP11A1: Cytochrome P450 11A1
CYP11B1/2: Cytochrome P450 11B
CYP17A1: Cytochrome P450 17A1
CYP21A2: Cytochrome P450 21A2
DZ: definitive zone
FZ: fetal zone
GW: gestational week
HFA: human fetal adrenal
HPA: hypothalamus-pituitary-adrenal
IHC: immunohistochemistry
MC2R: melanocortin 2 receptor
RT-PCR: real-time polymerase chain reaction
StAR: Steroidogenic acute regulatory protein
SULT2A1: Sulfotransferase Family 2A Member 1
TZ: translational zone
